# Understanding the responses of tillering to 2,4-D isooctyl ester in *Setaria viridis* L.

**DOI:** 10.1186/s12864-024-10579-6

**Published:** 2024-07-09

**Authors:** Wangdan Xiong, Xinfeng Jia, Qixin Wang, Nina Zhong, Hanchi Gao, Lingxin Zhang, Juan Sun

**Affiliations:** 1 Grassland Agri-Husbandry Research Center, College of Grassland Science, Qingdao Agricultural University, Qingdao, Shandong China; 2 Key Laboratory of National Forestry and Grassland Administration on Grassland Resources and Ecology in the Yellow River Delta, Qingdao Agricultural University, Qingdao, 266109 China; 3 Qingdao Key Laboratory of Specialty Plant Germplasm Innovation and Utilization in Saline Soils of Coastal Beach, Qingdao Agricultural University, Qingdao, 266109 China

**Keywords:** *Setaria viridis*, Tillering, Phytohormones, Gene expression, 2,4-D isooctyl ester

## Abstract

**Background:**

Green foxtail [*Setaria viridis* (L.)] is one of the most abundant and troublesome annual grass weeds in alfalfa fields in Northeast China. Synthetic auxin herbicide is widely used in agriculture, while how auxin herbicide affects tillering on perennial grass weeds is still unclear. A greenhouse experiment was conducted to examine the effects of auxin herbicide 2,4-D on green foxtail growth, especially on tillers.

**Results:**

In the study, 2,4-D isooctyl ester was used. There was an inhibition of plant height and fresh weight on green foxtail after application. The photosynthetic rate of the leaves was dramatically reduced and there was an accumulation of malondialdehyde (MDA) content. Moreover, applying 2,4-D isooctyl ester significantly reduced the tillering buds at rates between 2100 and 8400 ga. i. /ha. Transcriptome results showed that applying 2,4-D isooctyl ester on leaves affected the phytohormone signal transduction pathways in plant tillers. Among them, there were significant effects on auxin, cytokinin, abscisic acid (ABA), gibberellin (GA), and brassinosteroid signaling. Indeed, external ABA and GA on leaves also limited tillering in green foxtail.

**Conclusions:**

These data will be helpful to further understand the responses of green foxtail to 2, 4-D isooctyl ester, which may provide a unique perspective for the development and identification of new target compounds that are effective against this weed species.

**Supplementary Information:**

The online version contains supplementary material available at 10.1186/s12864-024-10579-6.

## Introduction

Green foxtail (*Setaria viridis* L.) is one of the most abundant and troublesome annual grass weeds in crop and alfalfa fields in China [1, 2]. Green foxtail belongs to the family Gramineae, with the characteristics of drought resistance [3]. Because of its strong adaptability and tillering ability, green foxtail is regarded as a common malignant weed [4]. It has been revealed that when the green foxtail density is above the threshold of 32 plants/m^2^, it causes loss rate of alfalfa yield in alfalfa fields [5]. Tillering is an important agronomic trait of Poaceae that greatly determines seed output [6]. For crops, such as cucumber and sorghum, strong tillering ability can increase food production [7, 8]. However, it will increase the production of weed seeds, accelerating their reproduction and improving the propagation coefficient [9]. Since weed infestation can cause losses of crop production due to the competition with the water, light, nutrient, or other resources, weed management is important for crop protection [10–12]. Thus, decreasing the number of tillering would be a strategy for controlling green foxtail.

Tillers are generally initiated by the establishment and elongation of tiller buds, which can be manipulated by phytohormones [13]. It is well-known that phytohormones like auxin, cytokinin, gibberellic acid (GA) and strigolactone (SL) play important roles in tillering of monocots and branching of dicots. Auxin is known as the first hormone to regulate shoot branching [14]. Removing the apex abolish the inhibition of axillary bud outgrowth and plants will start branching [15]. External application of auxin inhibit the growth of tiller buds in rice and wheat [16, 17]. Cytokinin plays a positive role in axillary bud outgrowth, and exogenous application of cytokinin promotes axillary bud outgrowth in plants [18]. GA can inhibit tillering, mainly due to its antagonistic interaction with cytokinin [18]. Exogenous SL supply has been reported to inhibit tiller or branch production by reducing the cytokinin content in the tiller or branch buds [19, 20]. Therefore, the interactions of auxin, cytokinin and SL control the regulation of tiller bud development [21, 22]. Thus, synthetic hormones would be a strategy for weeds control by decreasing plant tillers [23].

Synthetic auxins are used as growth regulators for yield improvement in agriculture, and as herbicides for weeds control [23]. The 2,4-dichlorophenoxy acetic acid (2,4-D) is one of the most widely used synthetic auxin herbicides to control weeds in pastures, especially for some Poaceae crops, such as wheat and maize [24, 25]. 2,4-D is developed during World War II and there are over 600 2,4-D products currently on the market including 2,4-D isooctyl ester [26]. It is absorbed through roots, stems, and leaves, and then it is translocated to the meristems of the plant, interfering with plant physiological processes [26, 27]. 2,4-D controls many broadleaf weeds and spare monocots, such as *Chenopodium album*, *Xanthium sibiricum*, and *Sonchus arvensis* [28]. For *Euphorbia heterophylla* to obtain 90% of control, the required dose was 3710 g a.e./ha [29]. *Digitaria insularis* and *Amaranthus hybridus* were more sensitive to the herbicide because 90% control was obtained with doses close to 755, and 269 g a.e./ha, respectively [29]. Monocots, particularly grasses, may perceive or respond differently to exogenous synthetic auxins compared to dicots [26]. It is proposed that there is a difference in vascular tissue structure between dicots and monocots, contributing to the selectivity of auxinic herbicide [26].

The 2,4-D has been reported to be used in controlling green foxtail growth, while many studies have recommended the use of clethodim and glyphosate to control green foxtail rather than 2,4-D [14, 17]. Little is understood regarding the effects of 2,4-D isooctyl ester on plant and tiller bud growth in green foxtail under different application concentrations. Moreover, the mechanism of different tillering responses of 2,4-D isooctyl ester at different concentrations is still unclear.

Therefore, the objectives of this study are to (i) evaluate the effect of 2,4-D isooctyl ester on the outgrowth of tiller buds in *S. viridis* and (ii) identify metabolic pathways and candidate genes involved in the diverse tillering sensitivities at different concentrations of 2,4-D isooctyl ester by transcriptomic analysis. Undoubtedly, the results will be highly valuable to further understand the regulatory mechanism of auxin on tillering growth and provide new ideas for solving the challenges of plant hormone selectivity for green foxtail and other grass weeds.

## Results

### Dose-dependent effects of 2,4-D isooctyl ester on plant growth in green foxtail

**Fig. 1:**
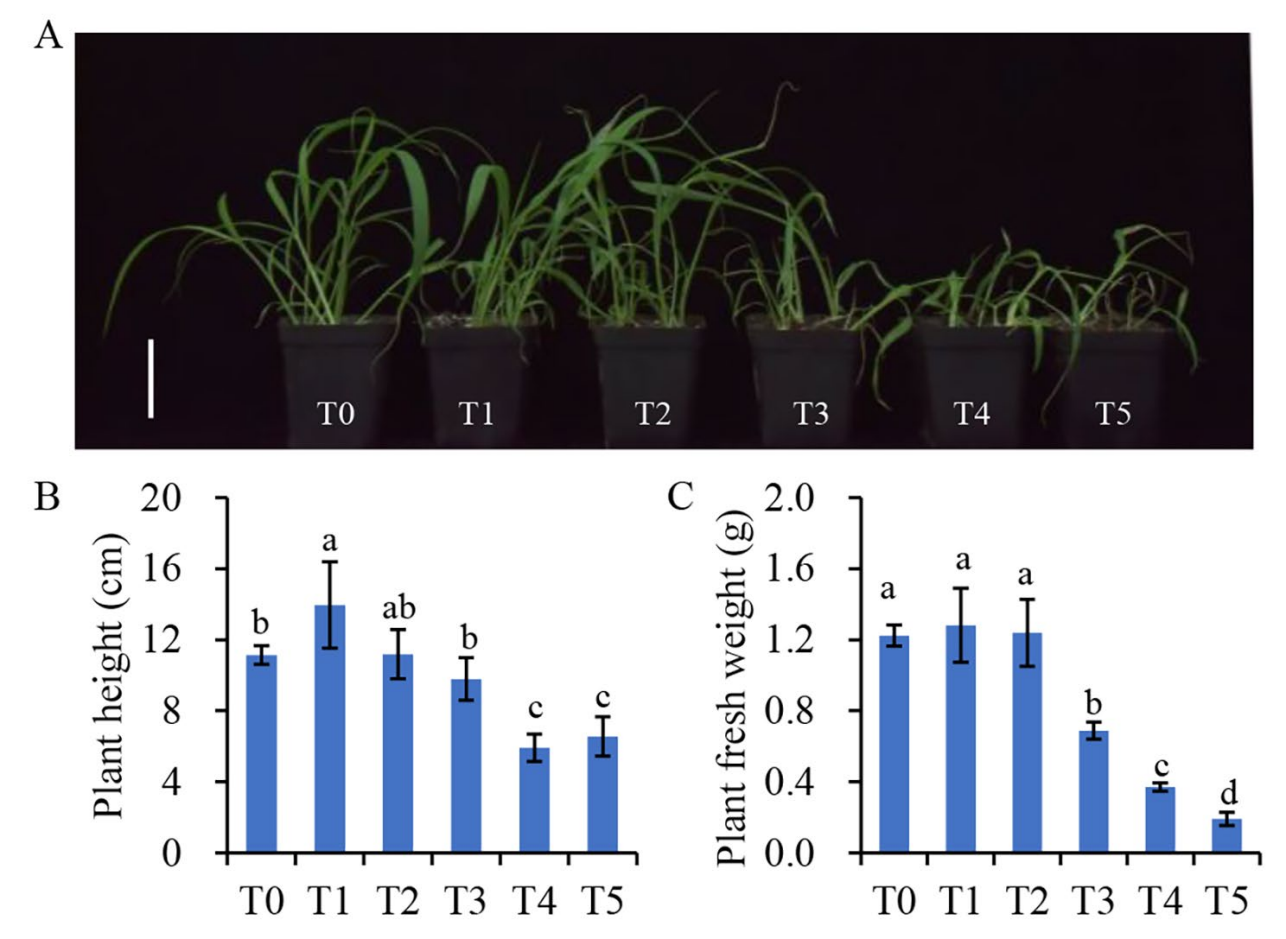
Effect of 2,4-D isooctyl ester on green foxtail after 7 days treatment. Plant phenotype (**A**), shoot height (**B**), and fresh weight (**C**) were measured after 7 days treatment. T0 to T5 represented concentrations of 0, 525, 1050, 2100, 4200, and 8400 g a.i./ha, respectively. The bar in Fig. 1A indicates 5 cm. Means followed by different lowercase letters are significantly different at *P* < 0.05

**Fig. 2:**
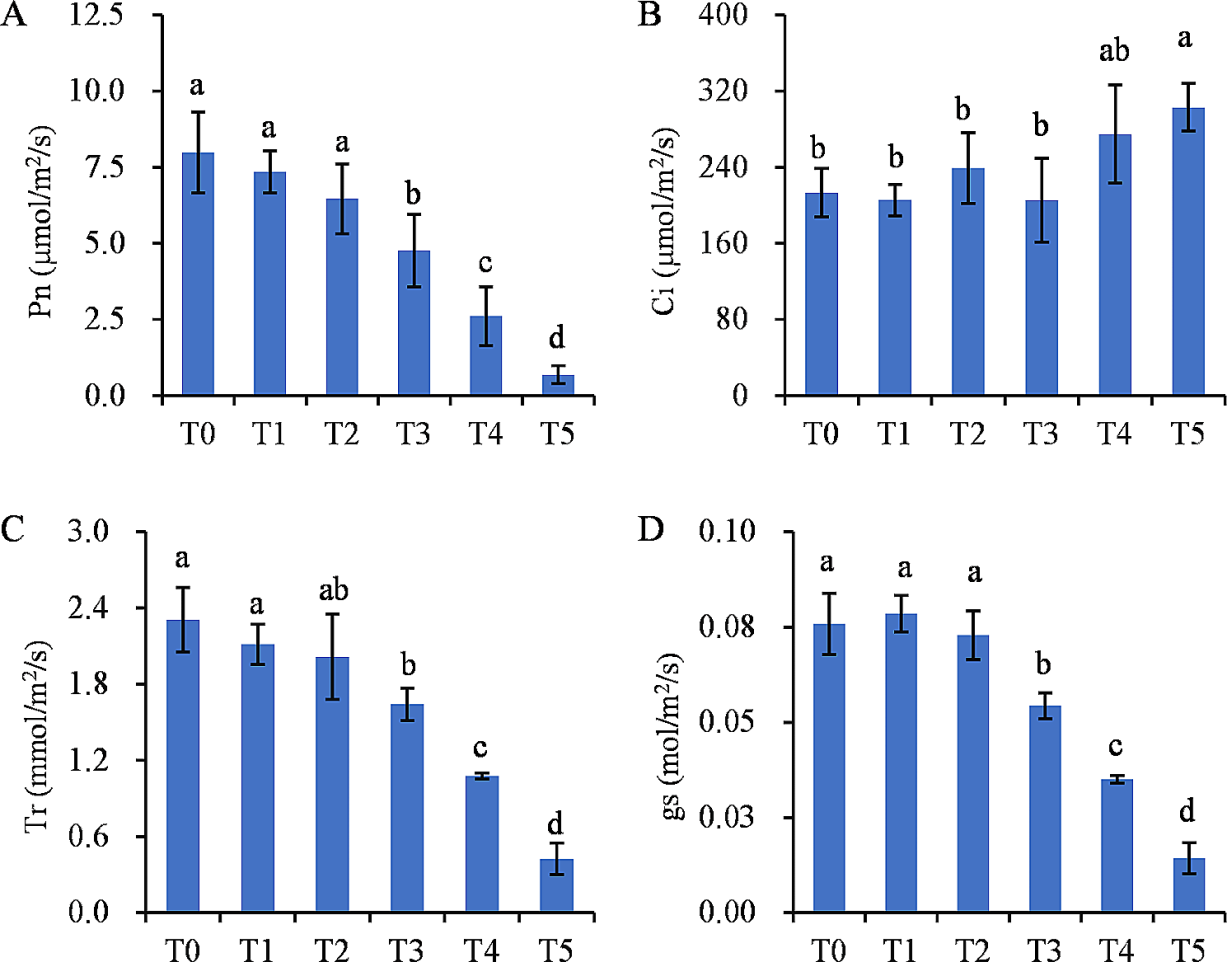
Effect of 2,4-D isooctyl ester on leaf net photosynthetic rate (**A**), intercellular CO_2_ concentrations (**B**), transpiration rate (**C**), and stomatal conductance (**D**) of green foxtail after 7 days treatment. T0 to T5 represented concentrations of 0, 525, 1050, 2100, 4200, and 8400 g a.i./ha, respectively. Pn, net photosynthetic rate; Ci, intercellular CO_2_ concentrations; Tr, transpiration rate; gs, stomatal conductance. Means followed by different lowercase letters are significantly different at *P* < 0.05

The objective of this study was to evaluate the effects of 2,4-D isooctyl ester on plant growth in green foxtail. In doing so, green foxtail was treated with different dosages of 2,4-D isooctyl ester and plant height, plant fresh weight, and leaf net photosynthetic rate were measured 7 days post-treatment. External 2,4-D isooctyl ester spraying had a significant effect on plant growth (*P* < 0.05, Fig. 1). The plant height increased and then decreased significantly after high-dose application of 2,4-D isooctyl ester (Fig. 1A and B). There was no significant effect on plant fresh weight at lower concentrations of 525 and 1050 g a.i./ha, but the plant fresh weight dramatically decreased at high concentrations (*P* < 0.05, Fig. 1B). Since part of the plant leaves were wilted and yellowed after treatment, the photosynthetic parameters were measured. The plant net photosynthetic rate (Pn) displayed a dramatic reduction under 2,4-D isooctyl ester treatment compared with the control, with the stomatal conductance (gs) and the transpiration rate (Tr) also reduced after treatment (*P* < 0.05, Fig. 2). Indeed, the malondialdehyde (MDA) content accumulated in the leaves of green foxtail after 2,4-D isooctyl ester treatment when the level was above 2100 g a.i. /ha (Additional file 1: Figure S1). The peroxidase (POD), catalase (CAT), and superoxide dismutase (SOD) enzyme activities were enhanced in all treatments (*P* < 0.05, Additional file 1: Figure S1).

### Effects of 2,4-D isooctyl ester on tiller production in green foxtail

**Fig. 3:**
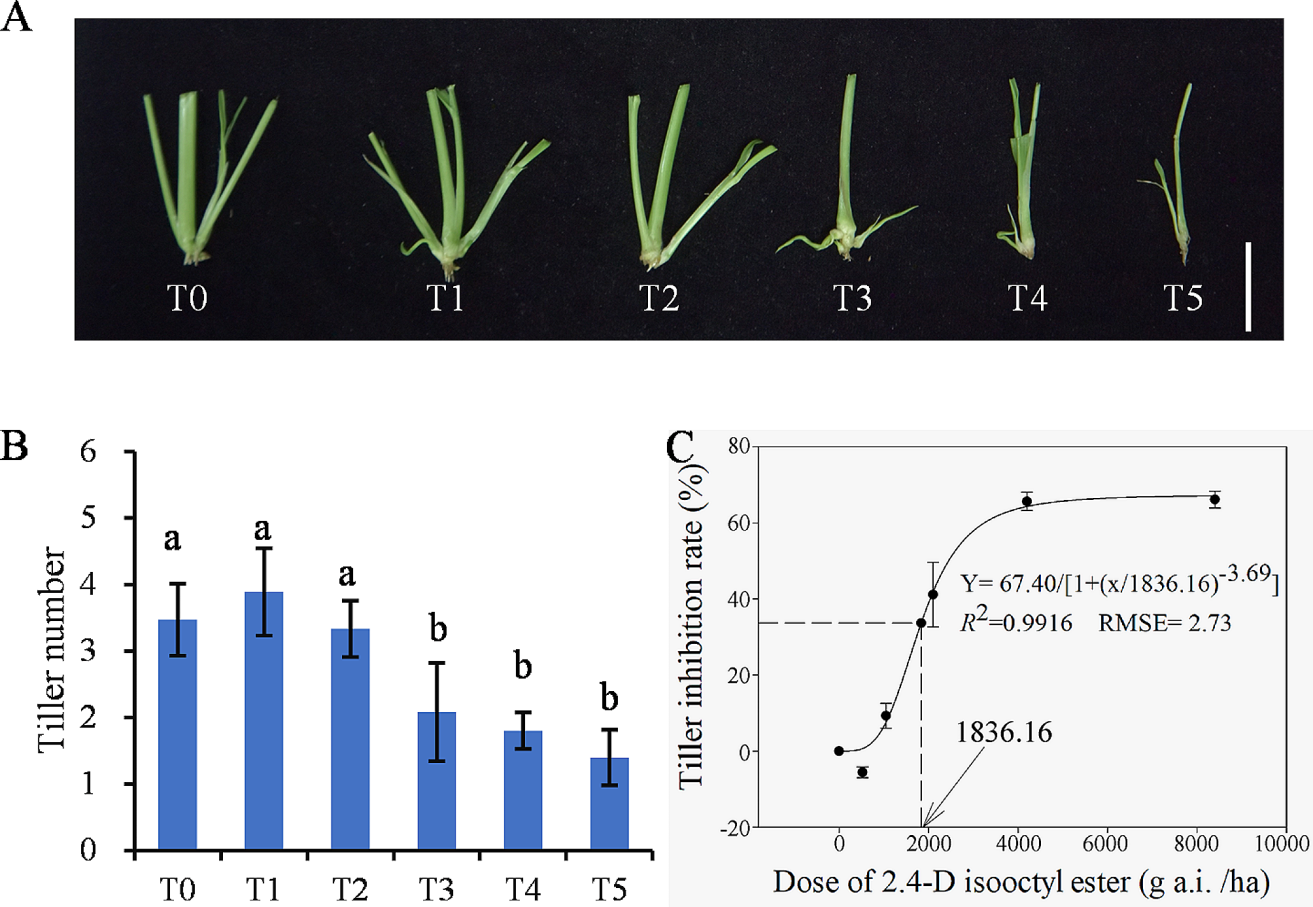
Effect of 2,4-D isooctyl ester on tillers in green foxtail after 7 days treatment. Plant tiller phenotype (**A**), tiller number (**B**), and dose response of tiller growth (**C**) were measured after 7 days treatment. T0 to T5 represented concentrations of 0, 525, 1050, 2100, 4200, and 8400 g a.i./ha, respectively. The bar in Fig. 3A indicates 2 cm. Means followed by different lowercase letters are significantly different at *P* < 0.05. The solid lines in Fig. 3C represent a three-parameter logistic model fitted to the data. Y (%) = 67.40/[1+ (x/1836.16)^−3.69^], R^2^ = 0.9916, RMSE = 2.73. Data represent the mean ± standard error of the mean (*n* = 30)

The 2,4-D isooctyl ester treatment affected not only the plant height and fresh weight but also the tiller number (Fig. 3). It had no significant effect on the tiller development at 7 days after treatment (DAT) under 525 and 1050 g a.i./ha compared to the control (Fig. 3A and B). When the dose was increased to 2100 g a.i./ha, it showed a significant inhibition effect on tiller number (Fig. 3A and B). The 2,4-D isooctyl ester dose required for 50% growth inhibition of tiller was estimated to be approximately 1836.16 g a.i./ha in green foxtail (Fig. 3C). Considering the 50% growth inhibition effect and the effects of 2,4-D isooctyl ester on plant growth, T4 was chosen for further analysis of effects of 2,4-D isooctyl ester on green foxtail tillers.

### Differentially expressed genes of 2,4-D isooctyl ester treatment on tiller in green foxtail

**Fig. 4:**
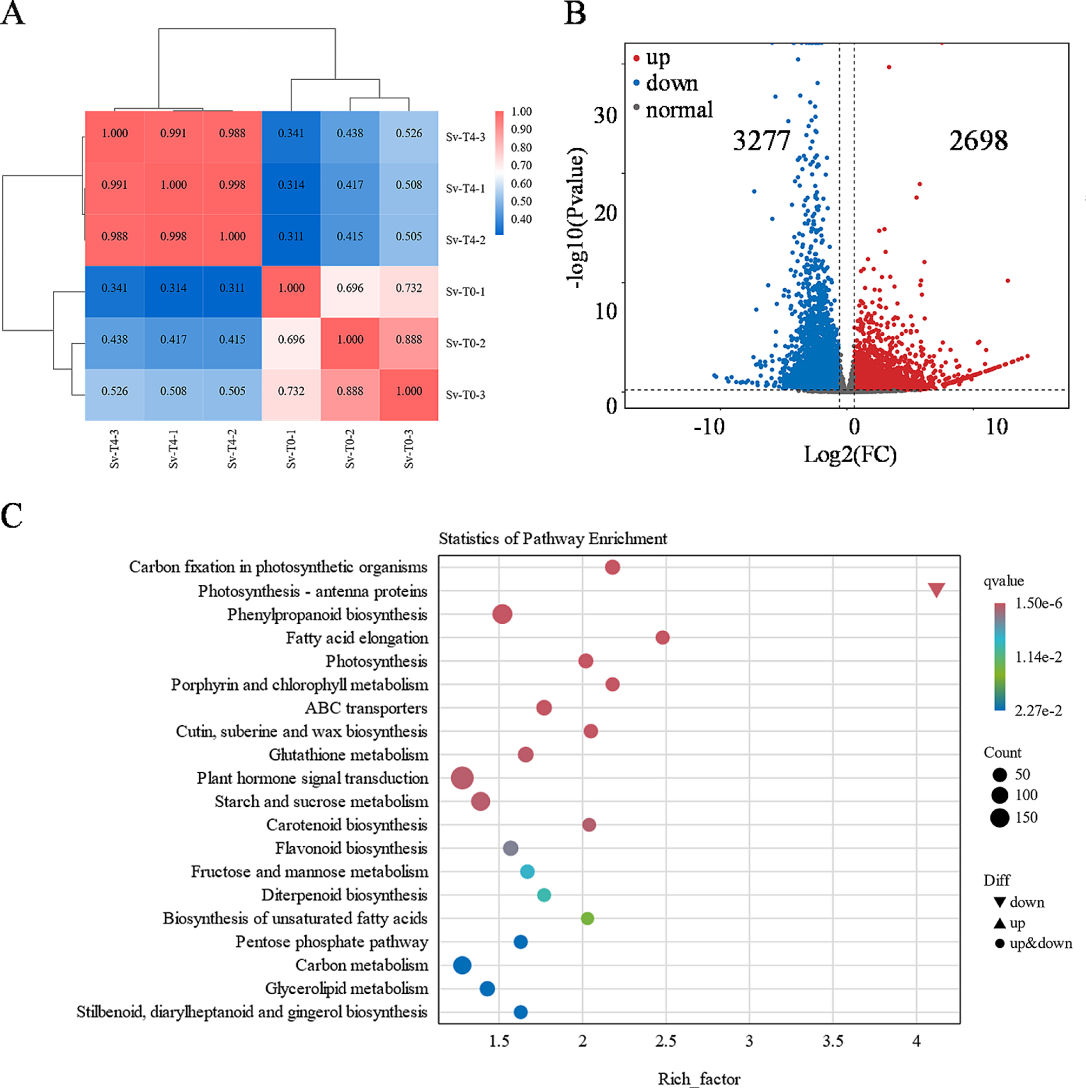
Pearson’s correlation (**A**), differentially expressed genes (**B**), and KEGG enrichment analysis (**C**) in green foxtail. Fold enrichment in a pathway means the ratio of the number of observed DEGs and expected DEGs. T0 and T4 represented concentrations of 0 and 2100 g a.i./ha, respectively

To analyze the 2,4-D isooctyl ester effects on tiller growth, tillers treated with 0 and 4200 g a.i./ha concentrations were used for transcriptome analysis. A total of 40.53 million reads to 56.80 million reads were obtained after RNA-Seq (Additional file 2: Table S1). Among them, 95.09–96.17% of the clean reads were successfully mapped to the green foxtail genome with a unique map ranging from 91.59 to 93.70% (Additional file 2: Table S1). The Pearson’s correlation coefficient heated map showed that samples under treatment clustered together (Fig. 4A). Based on the gene differential expression threshold of |log2fold change| ≥ 1 and false discovery rate (FDR) < 0.01, a total of 5975 differentially expressed genes (DEGs) were detected in tiller buds, of which 2698 DEGs were up-regulated and 3277 were down-regulated under treatment at 7 DAT (Fig. 4B; Additional file 1: Table S2).

### GO and KEGG Enrichment Analysis

To gain insights into the DEGs functional categories, gene ontology (GO) enrichment analysis was used to evaluate the specific functional role of all DEGs. The “cellular process” and “metabolic process” were the top GO terms in the “biological process” group. The “cellular anatomical entity”, “intracellular” and “protein-containing complex” were enriched in “cellular component” group ranked by gene number. The “binding”, “catalytic activity” and “transporter activity” categories were the most enriched GO terms in the “molecular function” group (Additional file 1: Figure S2).

To understand the functional enrichment classification of DEGs, KEGG analysis was carried out. The top 15 enriched pathways at 7 DAT including Carbon fixation in photosynthetic organisms, Photosynthesis-antenna proteins, Phenylpropanoid biosynthesis, Fatty acid elongation, Photosynthesis, Porphyrin and chlorophyll metabolism, and Plant hormone signal transduction (*q* < 0.05) (Fig. 4C). The plant net photosynthetic rate was reduced in the T4 (Fig. 2) and the soil and plant analyzer development (SPAD) was also reduced in the leaves (Additional file 1: Figure S3). Although the 2,4-D isooctyl ester was sprayed on the leaves, genes involved in plant hormone signal transduction accumulated after treatment. It was speculated that 2,4-D isooctyl ester might affect the plant hormone signal transduction and disturb the distribution and equilibrium of other plant hormones in the tillers.

### Differentially expressed genes analysis

**Fig. 5:**
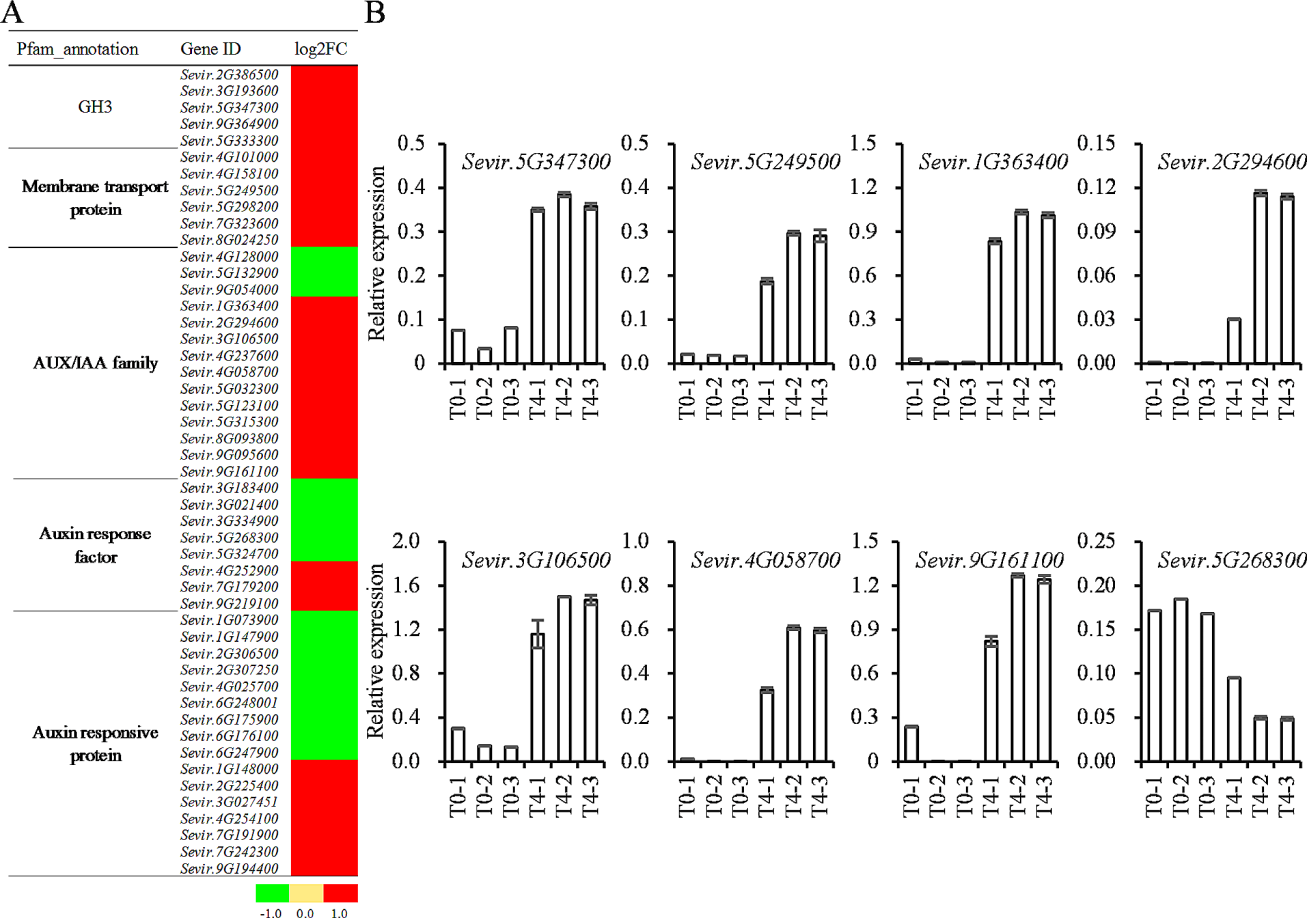
The expression level of DEGs related with auxin signal transduction in green foxtail. (**A**) DEGs related with auxin signal transduction in green foxtail log2(fold change) are shown. (**B**) Relative expression of DEGs associated in green foxtail by qRT-PCR. T0-1, T0-2, and T0-3 represented the control with three biological replicates and each was done with three technical replicates. T4-1, T4-2, and T4-3 represented the treatment with three biological replicates and each was done with three technical replicates

Given the above speculation, plant hormone metabolism and signal transduction pathways were further analyzed. DEGs were highly enriched in the plant hormone signal transduction pathways in tiller buds of green foxtail under treatment according to the KEGG enrichment analysis (Fig. 4C). A total of 49 DEGs related to auxin signal transduction pathway were identified (Fig. 5A). Among them, the expression levels of GH3 genes were highly enhanced after 2,4-D isooctyl ester treatment (Fig. 5A). Clearly, the expression levels of *auxin/indoleacetic acid* (*AUX/IAA*), *auxin response factor* (*ARF*), and *small auxin-up RNA* (*SAUR*) genes were also regulated after 2,4-D isooctyl ester treatment. To verify this result, nine DEGs related to auxin signal transduction were randomly selected and their expression levels were validated by qRT-PCR. Indeed, the RNA-seq DEGs also differentially expressed between treatments via qRT-PCR (Fig. 5B). Genes such as *Sevir.5G347300*, *Sevir.5G249500*, *Sevir.1G363400*, and *Sevir.2G294600* were up-regulated after treatment in RNA-seq analysis, and they were also up-regulated in RT-qPCR (Fig. 5). For gene *Sevir.5G268300*, it was down-regulated after treatment in both RNA-seq and RT-qPCR analysis (Fig. 5).

**Fig. 6:**
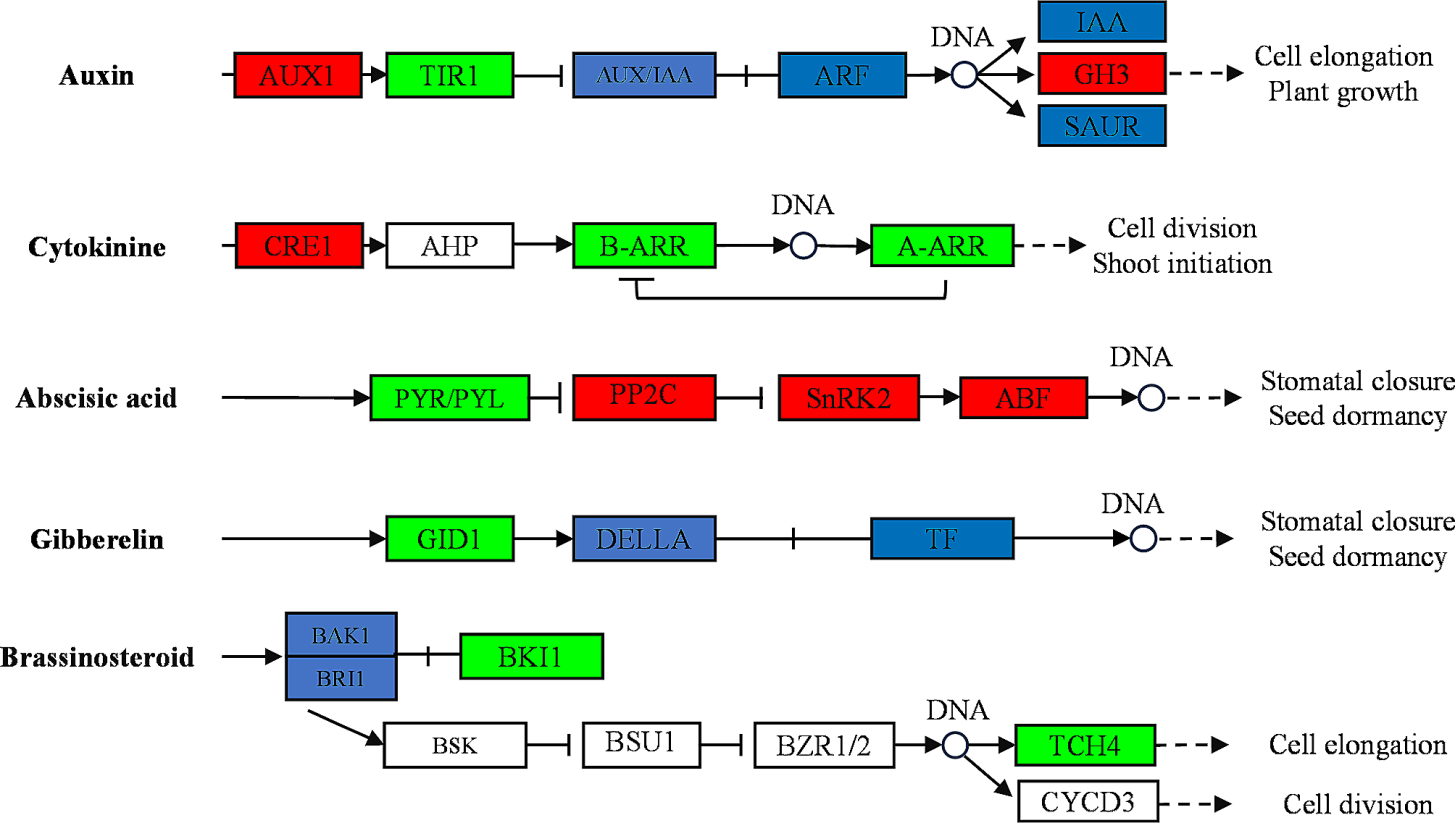
Map of the plant hormone signal transduction pathway. The red and green rectangles indicate the DEGs up-regulated and down-regulated in the 2, 4-D isooctyl ester treatment, respectively. The blue rectangle indicates that the DEGs containing both up-regulated and down-regulated

Similar results were also found in the cytokinin (CTK), abscisic acid (ABA), gibberellin (GA), and brassinosteroid (BR) synthesis and signal transduction pathways (Fig. 6; Additional file 1: Table S3). Two DEGs (*Sevir.1G319700* and *Sevir.4G202400*) annotated as cytokinin receptors (histidine kinase 2/3/4, AHK2_3_4) were up-regulated and cytokinin regulators encoding genes of cytokinin signaling response regulators Type-A and type-B were down-regulated. The genes encoding gibberellin receptor GID1 (*Sevir.2G045600*, *Sevir.2G395200*, *Sevir.2G422700*, and *Sevir.8G084700*) were down-regulated. Genes encoding abscisic acid receptor PYR/PYL family (*Sevir.3G213000*, *Sevir.7G137000*, and *Sevir.9G438800*) were down-regulated and their down-regulated genes encoding ABA responsive element binding factor (ABF; *Sevir.2G239700*, *Sevir.2G452300*, and *Sevir.4G081100*) were up-regulated. In the BR signal transduction pathway, *TCH4*, encoding a xyloglucan endo-transglycosylase, was down-regulated after 2,4-D isooctyl ester treatment. Therefore, it was speculated that the different impacts of 2,4-D isooctyl ester on tillering growth in green foxtail were related to different plant hormone metabolism and signal transduction responses.

MIR156 regulatory module is well believed to be involved in branching and tillering development in plant species [30]. Besides the above-mentioned genes in the plant hormone signal transduction pathways, transcriptomic analysis showed that six DEG belonging to SPL (SQUAMOSA PROMOTER BINDING PROTEIN-LIKE) family (*Sevir.2G276900*, *Sevir.4G200400*, *Sevir.2G336300*, *Sevir.2G336100*, *Sevir.3G023500*, and *Sevir.1G065500*) was down-regulated.

### Effects of phytohormones on tiller in green foxtail

**Fig. 7:**
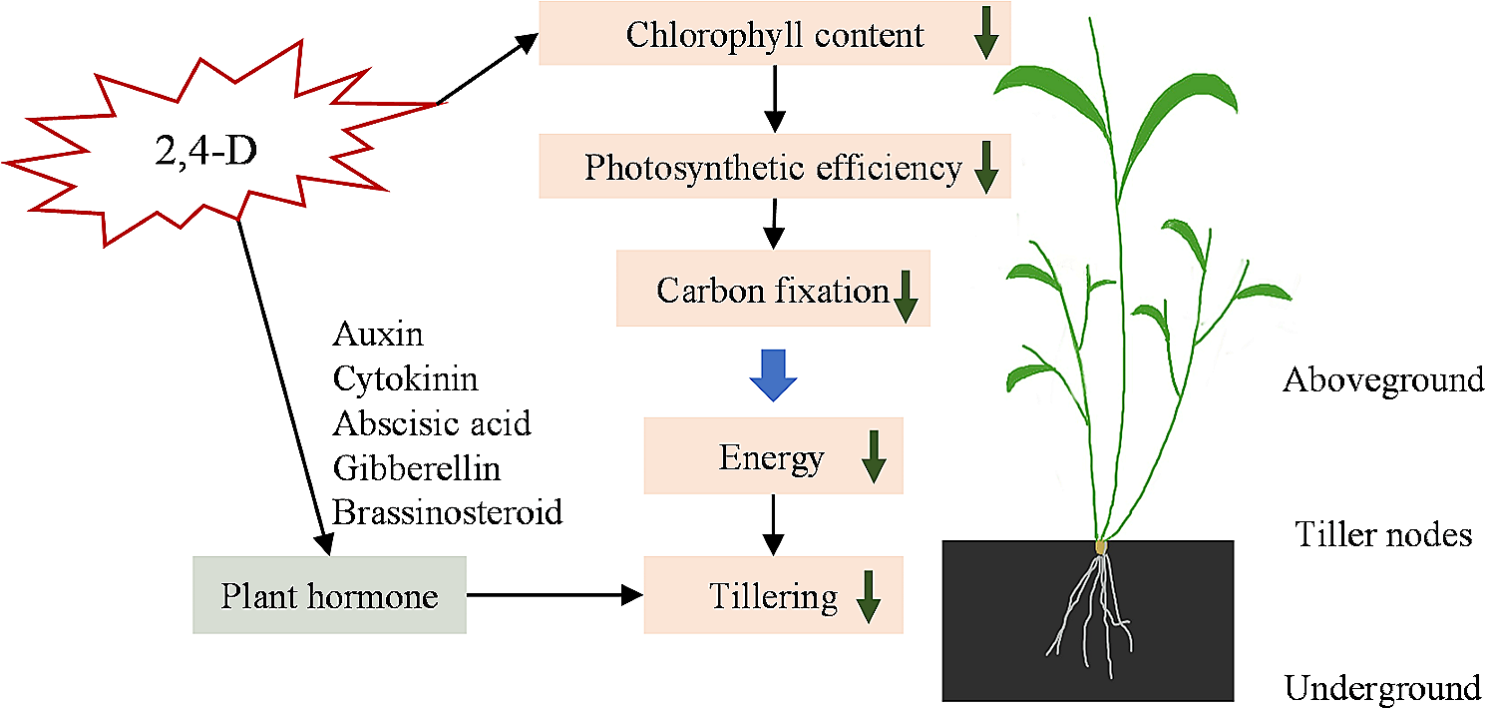
The regulatory network of green foxtail tillering response to 2,4-D isooctyl ester

Since genes involved in the phytohormones signal transduction pathways were regulated and the contents of ABA and tZR were changed in the tiller after 2,4-D isooctyl ester treatment, it was speculated that spaying other phytohormones besides auxin might also change tiller growth in green foxtail. To determine effects of different concentrations of other phytohormones (GA3, ABA, and 6-Benzylaminopurine) on tiller development, seedlings were foliar sprayed with 0, 300, 600, and 1200 mg/L. Indeed, spraying GA3 and ABA on leaves inhibited tiller growth (Additional file 1: Figure S4). But there was no significant effect after 6-BA application on tiller number (Additional file 1: Figure S4). Here, spraying 2,4-D isooctyl ester can affect the leaf chlorophyll content and reduce its photosynthetic efficiency, which might reduce plant carbon fixation (Fig. 7). On the other way, the treatments would affect the plant hormone distribution in the tillering buds. The reduced energy and varied plant hormone distribution affected the tiller growth (Fig. 7).

## Discussion

Synthetic hormones are used as growth regulators for improving crop yield in agriculture, and as herbicides for controlling weeds [23]. The 2,4-D is one of the most widely used synthetic auxin herbicides for the control of weeds in pastures, especially for some Poaceae crops for the control of broadleaf weeds and spare monocots [24, 25, 28]. But the effects of 2,4-D on green foxtail, especially on tillering, are unclear. Here, the effects on plant growth especially for the tiller bud were analyzed under different dose of 2,4-D isooctyl ester. Our study showed that high dose application of 2,4-D isooctyl ester inhibited plant growth and reduced the number of tiller buds significantly.

Tillering is controlled by genetic factors and environmental factors [31, 32]. Plant carbohydrate status is well known to affect tillering, and therefore reducing carbohydrate supply have been developed to account for how this factor modulates tillering under field conditions [33, 34]. Changes in photosynthetic leaf area affect the propensity of tiller buds for outgrowth in sorghum [35]. In the study, the experiment was conducted in pots with a relatively uniform potting medium, which could supply sufficient nutrient. Also, plants were grown with a same planting density in a controlled environment. Thus, planting density and nutrient scavenging were not the considered factors in the study. Sugars are necessary for the release of axillary buds for their apical dominance [36]. Indeed, in the study the SPAD value and photosynthetic efficiency were dramatically reduced. Application of 2,4-D isooctyl ester reduced the plant photosynthesis, thereby reducing the carbon fixation. The declined carbon fixation and limited energy supply might suppress the growth of tillers. Here, it is speculated that the photosynthesis of green foxtail after 2,4-D isooctyl ester treatment is weakened, unable to provide sufficient energy and produce less sugar for tiller bud development, which would partially explain the decrease in the number of tillers after the application of 2,4-D isooctyl ester.

Phytohormones also play important roles in tiller bud outgrowth. The regulation of plant lateral growth by auxin has been reported in various plant species, but simulative and suppressive effects have also been reported depending on the plant species and auxin concentration [16, 17, 28]. No differences in giant foxtail control were observed among herbicide application methods for glyphosate using either dicamba or 2,4-D isooctyl ester [28]. But the tiller buds are inhibited in crops such as rice and wheat after auxin was applied [16, 17]. Spraying indole-3-acetic acid (IAA) and naphthyl acetic acid (NAA, a synthetic auxin) on rice significantly restricted tiller bud elongation [16]. In wheat, tiller bud development was also completely inhibited by exogenous application of IAA [17]. Here, a relative high concentration of auxin on green foxtail would inhibit the tiller bud growth. Thus, application of synthetic auxin for weeds control depends on the species and the dosage.

Since the auxin pesticides were sprayed on leaves, it was unknown how it affected the tillering of green foxtail. Through the transcriptome data, the auxin transport and response genes were regulated after 2,4-D isooctyl ester application. Clearly, the expression levels of GH3, AUX/IAA, ARF, and SAUR genes were also regulated after 2,4-D isooctyl ester treatment, suggesting that auxin may participate in the regulation of tillering through influencing the balances of endogenous auxin metabolism and signaling. 2,4-D isooctyl ester can be moved throughout the plant through the phloem, and the reduction in tillers might cause by the variation of the phytohormone distribution in seedlings [28].

Besides the auxin response genes, other responses genes of phytohormones like CTK, GA, ABA, and BR were also regulated. Gene *TCH4*, encoding xyloglucan endotransglucosylase, was down-regulated in tillers after 2,4-D isooctyl ester application. BR is also a typical hormone controlling tiller initiation and there is a cross talk between auxin and BR signaling pathways [37–39]. *TCH4* can be regulated by BR because the promoter region of *TCH4* contains cis-regulatory element e-boxes binding to the transcription factor BES1 in the BR signaling pathway [40]. Genes involved in auxin were significantly up-regulated in plant roots under BR treatment [37, 39]. CTK plays a positive role in axillary bud outgrowth and the CTK-mediated genes *A-ARR* have been reported to be required for bud release in *Arabidopsis* [41, 42]. Exogenous application CTK promotes axillary bud outgrowth, which is accompanied by increases in CK content in buds or adjacent nodes [18]. The CTK signal transduction genes, *A-ARR* and *B-ARR*, were found to be down-regulated after 2,4-D isooctyl ester application, which indicated that the application of 2,4-D isooctyl ester could influence genes involved in CTK. Moreover, *A-ARR* may be the key factor in CTK signal transduction contributing to SLs-induced bud inhibition. SL represses auxin transport from the buds, thus inhibiting bud outgrowth whereas auxin up-regulates SL production to control apical dominance [19, 43]. Moreover, SLs can suppress the *A-ARR* expression independent of CTK signal transduction. ABA is associated with bud dormancy through an auxin-independent mechanism [44]. Therefore, application 2,4-D isooctyl ester on the leaves affected the expression of several hormone signal transduction genes and might affect distribution of phytohormone levels in tillers. In addition to the hormone signal transduction genes, it is generally believed that the MIR156-SPL module participates in the regulation of plant architecture, including tillering [30]. In treated green foxtail plants, the expression levels of six *SPL* genes differed from those in untreated plants. It is speculated that variation of distribution of hormone levels would be another main reason affecting the tillering of green foxtail after 2,4-D isooctyl ester treatment.

## Conclusion

Tiller production is important for grass weeds control. The 2,4-D isooctyl ester is one of the most widely used synthetic auxin herbicides to control weeds in pastures. However, how 2,4-D isooctyl ester affects tillering in grass weeds is still unclear. This study aimed to elucidate the roles and the underlying mechanisms of auxin in regulating tiller development. Green foxtail seedlings were treated with different concentrations of 2,4 isooctyl ester and dose-dependent inhibitory effects on tiller production were observed. Auxin-inhibition of tillering was mainly due to its effect on inhibition of photosynthesis and the distribution of other plant hormones, as shown by the changed plant hormone signal transduction expression in 2,4 isooctyl ester treated plants. Furthermore, auxin could act through regulating the expression of SPL specifically expressed in axillary buds to induce bud outgrowth. These results provide insights into the regulatory mechanisms of auxin for tiller bud outgrowth through crosstalk with phytohormones.

## Materials and methods

### Plant Material, growth conditions

Wild-type A10.1 seeds of green foxtail (*S. viridis*) were germinated in petri dishes for seven days. After emergence, green foxtail seedlings were sown to six uniform seedlings per pot containing a mixture of soil, vermiculite, and organic fertilizer (3:1:1, v/v/v) and grown under greenhouse conditions at a temperature of 25 °C, with a 16 h/8 h photoperiod at a relative humidity of 65%. The plants were watered weekly with Hoagland solution.

### 2,4-D isooctyl ester treatment

2,4-D isooctyl ester 50% emulsifiable concentrate (EC) was provided by Shandong Zhongshi Pharmaceutical Co., Ltd. (Liaocheng, China). Twenty-five-day-old seedlings, reached the 3- to 4-leaf stage and the length of the first tiller bud was less than 0.5 cm, were treated with 2,4-D isooctyl ester. Based on the recommended concentration approximately 500 to 1000 g a.i./ha (a.i., active ingredient), the treatments consisted of applications of the herbicide 2,4-D isooctyl ester at doses of 0, 525, 1050, 2100, 4200, and 8400 g a.i./ha. Deionized water spray was used as a control. Spraying was performed using a 3WP-2000 Walking Spray Tower (Nanjing Institute of Agricultural Mechanization, Ministry of Agriculture and Rural Affairs, Nanjing, China). The tiller buds were measured in 30 seedlings per treatment at 7 DAT, and the experiment was conducted in three independent replicates.

### External application of ABA, GA3, and 6-BA

To ascertain the effects of exogenous ABA, GA and CTK on tiller growth, ABA (catalog number A8060, Solarbio, China), GA3(catalog number G8910, Solarbio, China), and 6-BA (catalog number A8170, Solarbio, China) were applied to green foxtail. Spraying was performed using a 3WP-2000 Walking Spray Tower (Nanjing Institute of Agricultural Mechanization, Ministry of Agriculture and Rural Affairs, Nanjing, China). Uniform 20-day-old seedlings with the length of the first tiller bud less than 0.5 cm were treated with ABA, GA3 or 6-BA at concentrations of 300, 600, and 1200 mg/L. Deionized water was applied as a control. Plants were sprayed twice with a 3WP-2000 Walking Spray Tower. Each treatment included five pots containing six seedlings each, and the experiment was repeated three times.

### Measurement of Leaf photosynthetic rate and measurement of enzyme activity assay

The photosynthetic rate of the uppermost expanded leaves was measured with an LI-6800 portable photosynthesis system at 7 DAT. The photosynthetically active radiation was set to 800 µmol/m^2^/s. The freeze-dried and powered leaf samples were used to analyze the content of MDA and enzyme assay activities of CAT, POD, and SOD [45].

### Sample Preparation for RNA sequencing, qPCR validation

Tiller buds of 1–2 cm were collected at 7 DAT and then immediately frozen in liquid nitrogen and stored at − 80 °C and total RNA was extracted with the plant RNA kit (catalog number RC411-01, Vazyme, China) according to the manufacturer’s instruction. RNA concentration and quality were assessed using a NanoDrop2000 and an Agilent 2100. The mRNA was obtained by enriching qualified RNA using magnetic beads equipped with oligo (dT). The library was constructed and the quality of the constructed libraries was strictly monitored before sequencing with an Illumina NovaSeq 6000.

A total of 1 µg RNA was used for the synthesis of first-strand cDNA for qRT-PCR (catalog number R323-01, Vazyme, China). qPCR was carried out according to MIQE guidelines with SYBR Green (Takara, Tokyo, Japan) using the CFX96 (Bio-Rad, USA). The *β-actin* gene *Sevir.7G305900* was used as the reference gene to normalize the target gene expression levels in green foxtail and the 2^−ΔΔCT^ method was used to calculate the gene expression level [46]. All qPCR primers are shown in Table S4.

### Differential expression and Gene Ontology enrichment analysis

Quality control of raw reads was performed to remove adapter sequences and clean reads were mapped against the *Setaria viridis* reference genome (*Setaria viridis* v4.1, Phytozome) using HISAT2 software (version 2.1.0) [47]. For quality control, raw data were filtered by removing the reads with adapter pollution, unknown nucleotides more than 5%, or Q20 value ≤ 20% (percentage of sequences with sequencing error rates < 1%), and the remaining highly qualitative clean reads were then subjected to further analyses. Gene expression levels were estimated using fragments per kilobase of exon per million fragments mapped (FPKM) values by the Cufflinks software. The comparison of gene expression differences was carried out between treatment (T4) and control (T0) based on the ratio of FPKM values using the DESeq2 software (version 1.22.1) [48]. GO annotation was obtained using Blast2 GO program (version 3.0.8) at a threshold e-value ≤ 1e^–5^ [49]. All DEGs were subjected to GO enrichment analysis using the GOseq R package based on FDR and fold change (FC) of genes betweenT4 and T0, with a level of | log2FC| ≥ 1 and FDR < 0.05 as the threshold. Additionally, Kyoto Encyclopedia of Genes and Genomes (KEGG) pathway annotation was obtained using KEGG Automatic Annotation Server (KAAS) and KEGG analysis was also performed on the DEGs using cluster Profiler R packages to find KEGG pathway [50, 51].

### Statistical analysis

All experiments were performed with three replicates, and the means and standard deviations were calculated. ANOVA was employed to evaluate the effect of external treatment with 2,4-D isooctyl ester using IBM SPSS software. Differences were distinguished by LSD test at the 0.05 probability level.

The tiller bud inhibition rate collected was modelled using logistic models. SigmaPlot (Version 11, Sysat Software, Inc., Point Richmond, CA, USA) was used to perform nonlinear regression analysis to determine how inhibition rate was affected by 2,4-D isooctyl ester dosage. The goodness-of-fit of models was assessed according to their R^2^ and root mean-square error (RMSE) values. Nonlinear regression analysis was applied to calculate the effect of 2,4-D isooctyl ester concentration on tiller inhibition rate analysis with the following functional three-parameter logistic model:

Y = A/[1+(x/x50)^B^]

Here, Y is the percentage (%) of inhibition rate under different concentration of 2,4-D isooctyl ester treatments, A is the maximum germination or emergence (%), x50 is the 2,4-D isooctyl ester dosage required for 50% of the highest inhibition rate and B represents the slope [52].

### Electronic supplementary material

Below is the link to the electronic supplementary material.

Supplementary Material 1

Supplementary Material 2

## Data Availability

Sequence data that support the findings of this study have been deposited in NCBI SRA BioProject with accession number of PRJNA1111665.

## Abbreviations

2,4-D: 2,4-dichlorophenoxy acetic acid
MDA: Malondialdehyde
ABA: Abscisic acid
GA: Gibberellic acid
SL: Strigolactone
Pn: net photosynthetic rate
Gs: stomatal conductance
Tr: Transpiration rate
POD: peroxidase
SOD: superoxide dismutase
CAT: catalase
DAT: Days after treatment
FDR: false discovery rate
DEG: Differential expression gene
GO: Gene Ontology
SPAD: Soil and plant analyzer development
AUX: Auxin
IAA: Indole-3-acetic acid
AFR: Auxin response factor
SAUR: Small auxin-up RNA
CTK: Cytokinin
BR: Brassinosteroid
ABF: element binding factor
tZR: trans-Zeatin Riboside
6-BA: 6-Benzylaminopurine
NAA: naphthyl acetic acid
FPKM: Fragments per kilobase of exon model per million mapped reads
FC: fold change
KEGG: Kyoto Encyclopedia of Genes and Genomes
KAAS: KEGG Automatic Annotation Server
RMSE: Root mean-square error
