# Supplementary material for: Understanding the responses of tillering to 2,4-D isooctyl ester in Setaria viridis L
Source: BMC Genomics. 2024 Jul 9;25:682. doi: 10.1186/s12864-024-10579-6 (PMC11232182; doi:10.1186/s12864-024-10579-6)
Supplement: Supplementary file 1 — Supplementary Material 1 [file 12864_2024_10579_MOESM1_ESM.docx]

**Additional Files:**

**Additional file 1: Figure S1.** The MDA content, CAT activity, SOD activity, and POD activity influenced by 2,4-D application in green foxtail leaves, with water as control.

**Additional file 1: Figure S2.** GO enrichment annotation of differentially expressed genes in green foxtail tiller after 2,4-D application.

**Additional file 1: Figure S3.** The effect of 2,4-D on SPAD of green foxtail leaves.

**Additional file 1: Figure S4.** The effects of external spraying of abscisic acid (A), Gibberellic acid (B) and 6-benzyl aminopurine (C) on tiller bud outgrowth in green foxtail.

Figure S1. The MDA content, CAT activity, SOD activity, and POD activity influenced by 2,4-D application in green foxtail leaves, with water as control.

Different low case letters above columns indicate statistical differences at *P* ≤ 0.05.

Figure S2. GO enrichment annotation of differentially expressed genes in green foxtail tiller after 2,4-D application.

Figure S3. The effect of 2,4-D on SPAD of green foxtail leaves.

Different low case letters above columns indicate statistical differences at *P* ≤ 0.05.

Figure S4. The effects of external spraying of abscisic acid (A), Gibberellic acid (B) and 6-benzyl aminopurine (C) on tiller bud outgrowth in green foxtail.

Error bars represent the mean ± SD (n = 6). Different letters represent significant differences (*P* < 0.05).
